# Persistence of Microcystin in Three Agricultural Ponds in Georgia, USA

**DOI:** 10.3390/toxins16110482

**Published:** 2024-11-07

**Authors:** Jaclyn E. Smith, James A. Widmer, Jennifer L. Wolny, Laurel L. Dunn, Matthew D. Stocker, Robert L. Hill, Oliva Pisani, Alisa W. Coffin, Yakov Pachepsky

**Affiliations:** 1 Environmental Microbial Food Safety Laboratory, Agricultural Research Service, United States Department of Agriculture, Beltsville, MD 20705, USA; jackie.smith@usda.gov (J.E.S.); matthew.stocker@usda.gov (M.D.S.); 2 Department of Environmental Science and Technology, University of Maryland, College Park, MD 20742, USA; rlh@umd.edu; 3 Oak Ridge Institute for Science and Education, Oak Ridge, TN 37830, USA; 4 Department of Food Science and Technology, University of Georgia, 100 Cedar Street, Athens, GA 30602, USA; james.widmer@uga.edu (J.A.W.); laurel.dunn@uga.edu (L.L.D.); 5 Office of Regulatory Science, Center for Food Safety and Applied Nutrition, US Food and Drug Administration, College Park, MD 20740, USA; jennifer.wolny@fda.hhs.gov; 6 Southeast Watershed Research Laboratory, Agricultural Research Service, United States Department of Agriculture, Tifton, GA 31793, USA; oliva.pisani@usda.gov (O.P.); alisa.coffin@usda.gov (A.W.C.)

**Keywords:** microcystin, cyanotoxin, cyanobacteria, irrigation ponds, water quality, livestock ponds, agricultural ponds, monitoring

## Abstract

Cyanobacteria and their toxins can have multiple effects on agricultural productivity and water bodies. Cyanotoxins can be transported to nearby crops and fields during irrigation and may pose a risk to animal health through water sources. Spatial and temporal variations in cyanotoxin concentrations have been reported for large freshwater sources such as lakes and reservoirs, but there are fewer studies on smaller agricultural surface water bodies. To determine whether spatiotemporal patterns of the cyanotoxin microcystin occurred in agricultural waters used for crop irrigation and livestock watering, three agricultural ponds on working farms in Georgia, USA, were sampled monthly within a fixed spatial grid over a 17-month period. Microcystin concentrations, which ranged between 0.04 and 743.75 ppb, were determined using microcystin–ADDA ELISA kits. Temporal stability was assessed using mean relative differences between microcystin concentrations at each location and averaged concentrations across ponds on each sampling date. There were locations or zones in all three ponds that were consistently higher or lower than the average daily microcystin concentrations throughout the year, with the highest microcystin concentrations occurring in winter. Additionally, microcystin patterns were strongly correlated with the patterns of chlorophyll, phycocyanin, and turbidity. The results of this work showed that consistent spatiotemporal patterns in cyanotoxins can occur in produce irrigation and livestock watering ponds, and this should be accounted for when developing agricultural water monitoring programs.

## 1. Introduction

Harmful algal blooms commonly occur in many freshwater sources and are projected to become more frequent and more intense due to climate change [1,2]. When toxic cyanobacteria are the main component of these blooms, in which case they are called cyanoHABs, negative effects on aquatic organisms, birds, animals, and human health are likely [3,4]. Irrigation of crops with water affected by toxin-producing cyanoHABs can lead to the accumulation of cyanotoxins in edible parts of plants [5,6]. Livestock illness and death resulting from the consumption of cyanotoxin-laden waters are well documented [7,8,9,10,11]. Microcystin (MC) is the most common and pervasive cyanotoxin; the MC congeners MC-LR, MC-LA, MC-RR, and MC-YR are the most prevalent and are considered environmental and human health hazards [12,13]. The World Health Organization has established suggested guideline values for MC concentrations in drinking and recreational waters [14] but not in agricultural-use waters. The environmental and physiological conditions leading to toxin production are unknown or unpredictable for most toxin-producing cyanobacterial species, thereby requiring routine sampling and multi-faceted monitoring programs to safeguard environmental and human health [15,16].

MC concentrations are highly variable in time and space. The spread of MC concentrations across a water body may reach a difference of four orders of magnitude [17]. The variability of both cell and toxin concentrations is particularly evident when considering surface scum formations that often occur in lacustrine environments [18,19]. Consequently, monitoring strategies that take into account the potential for scum formation are better at predicting the presence and concentration of cyanobacteria and cyanotoxins [19,20,21]. Concurrently, there are detailed reports of the persistence of MCs within waters and sediments seasonally [22,23] and annually [24,25]. These studies have characterized cell and toxin concentrations in larger bodies of water, but this type of characterization in the smaller water bodies that are often used in agricultural irrigation operations is lacking.

In the Southeastern United States, most produce farming operations are conducted spring through fall, while livestock operations are a year-round activity [26]. These activities require a steady supply of safe irrigation water that can augment the variable rain events in this region [27]. Surface waters used to provide water to agricultural operations are closely monitored for microbial water quality, but most often, cyanobacteria and cyanotoxins are not considered alongside other contributing factors (e.g., rainfall, temperature, physiochemical parameters, and seasonality) that can degrade water quality [28,29]. In Georgia, the climate shift to warm winters and hot summers, coupled with a rapidly urbanizing landscape, is increasing the susceptibility of waters to cyanoHABs and their toxins [30,31], all while water use for agriculture activities has increased due to expanding broiler chicken, peanut, and cotton farming operations alongside more frequent and prolonged droughts [32]. Haynie et al. [33] reported on livestock and wildlife mortalities associated with livestock drinking water ponds in Georgia that were experiencing *Microcystis* blooms. More recently, Mohamed et al. [34] reported on the combined effects of livestock herds being exposed to MCs through drinking water and alfalfa that had been irrigated with MC-laden waters. Thus, the need exists to merge the modeling of cyanobacterial blooms and cyanotoxins that have been outlined for large water bodies with the frequently used small water bodies found in agricultural settings.

Spatially rigorous surface water sampling has demonstrated the existence of persistent patterns in MC concentrations in large water bodies. In 2016 and 2017, the spatial MC distributions in Lake Erie, one of the Great Lakes on the US–Canada border, were comparable, but the timing and concentrations were different [35]. Notably, MC concentrations were higher in nearshore locations than in the center of the lake [35]. Similar observations have been made regarding MC and other cyanotoxins in the other Great Lakes [36,37,38]; Upper Klamath Lake, Oregon, USA [16]; Lakes Pouyang [17] and Taihu [23,25], China; Lake Biwa, Japan [39]; and numerous reservoirs in Brazil [40]. While the Southeastern region of the US has been determined to be most at risk for cyanobacterial blooms, the EPA’s National Lake Assessment program only assessed data from water bodies > 40,500 m^2^ in size [41]. In Georgia, an estimated 100,000 ponds exist to irrigate 1.3 million acres of farmland [42,43]. However, information regarding the persistence of MC in both fluvial and lacustrine systems is lacking [44], especially for the region’s smaller, shallow water bodies [31], which are increasingly being used for agriculture operations [42,45].

Work conducted previously by this research group demonstrated that cyanobacterial populations can be temporally and spatially stable in small agricultural ponds (<8000 m^2^) and monitored using simple water quality parameters, such as turbidity and colored dissolved organic matter [46,47]. However, these relationships appear to be site-specific as other studies have found different correlations between cyanobacterial populations and their toxins and water quality parameters (e.g., chlorophyll, pH, and temperature [16]; phosphorus and phycocyanin [48]; and chlorophyll only [49]). Here, we capitalize on these sampling strategies developed for small agricultural ponds to characterize the year-round presence of cyanobacteria and cyanotoxins in three agricultural ponds that provide irrigation waters for crops and drinking water for livestock in Georgia, USA, and determine whether relationships between MC concentrations and certain water quality variables exist if persistent spatial patterns of MC are identified.

## 2. Results

### 2.1. Data Summary

#### 2.1.1. Weather Data

Weather data were collected from weather stations within 1 km of all three ponds. Due to the proximity (0.5 km) of Pond 2 and Pond 3, the same weather station was utilized for both ponds. Average daily air temperature and daily precipitation data are displayed in Supplemental Figure S1 for Pond 1 and Supplemental Figure S2 for Ponds 2 and 3. The average hourly air temperatures during sampling at Pond 1 ranged from 0.345 to 33.47 °C throughout the study period. The total accumulated rainfall over the study period at Pond 1 was 1333 mm. No precipitation was recorded between 12 August 2022 and 16 October 2022. At Ponds 2 and 3, the average hourly air temperatures during sampling ranged from 2.65 to 33.74 °C throughout the study period. Similar to Pond 1, Ponds 2 and 3 experienced a period with little to no rainfall from 10 September 2022 to 12 October 2022. The total accumulation of precipitation at Ponds 2 and 3 over the study period was 1764 mm. All three ponds exhibited seasonal variations in air temperature, with the winter months having lower air temperatures than the summer months.

#### 2.1.2. Microcystin Concentrations

**Figure 1:**
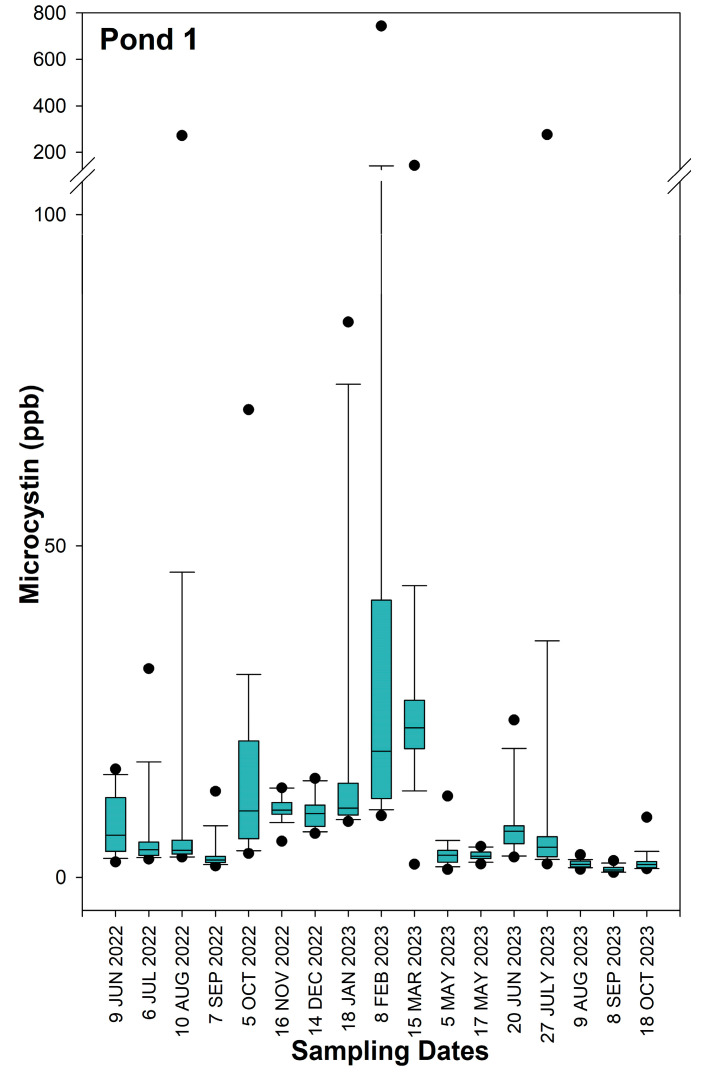
Box plots of microcystin concentrations in Pond 1 over the 17-month study period. Medians are displayed with solid lines. The upper and lower sides of the box represent the 1st and 3rd quartiles, with the distance between the quartiles being the interquartile range of microcystin concentration distributions for each sampling day. The whiskers show the minimum and maximum values that are not considered outliers. The upper and lower dots represent outliers, which are calculated as values > 3rd quartile + 1.5 × interquartile range or <1st quartile − 1.5 × interquartile range, respectively.

**Figure 2:**
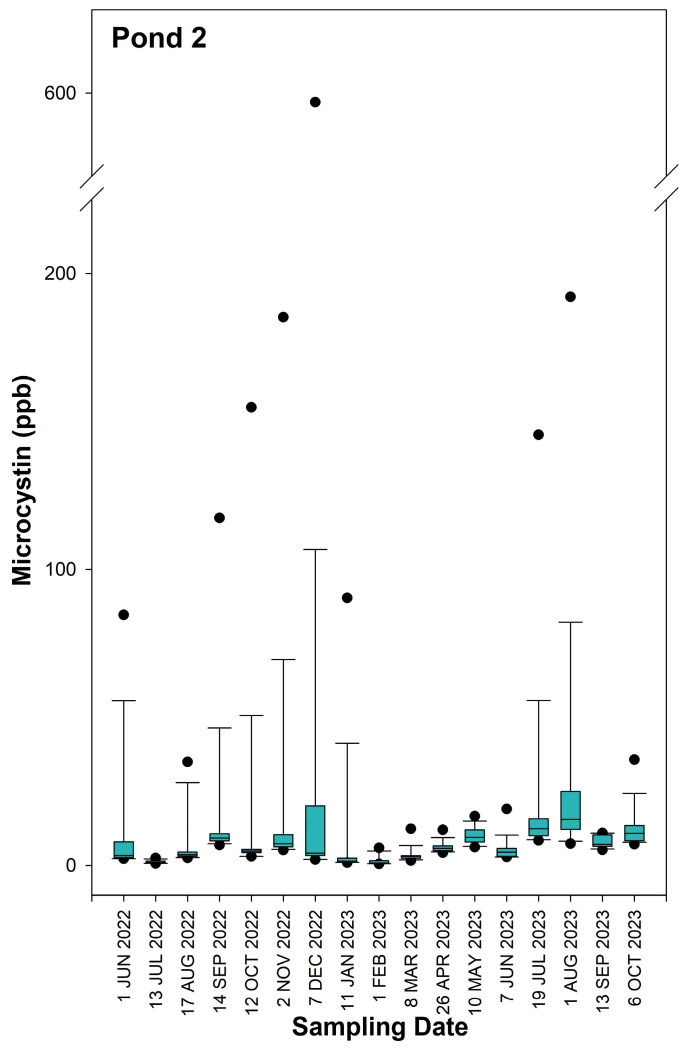
Box plots of microcystin concentrations in Pond 2 over the 17-month study period. Medians are displayed with solid lines. The upper and lower sides of the box represent the 1st and 3rd quartiles, with the distance between the quartiles being the interquartile range of microcystin concentration distributions for each sampling day. The whiskers show the minimum and maximum values, that are not considered outliers. The upper and lower dots represent outliers, which are calculated as values > 3rd quartile + 1.5 × interquartile range or <1st quartile − 1.5 × interquartile range, respectively.

**Figure 3:**
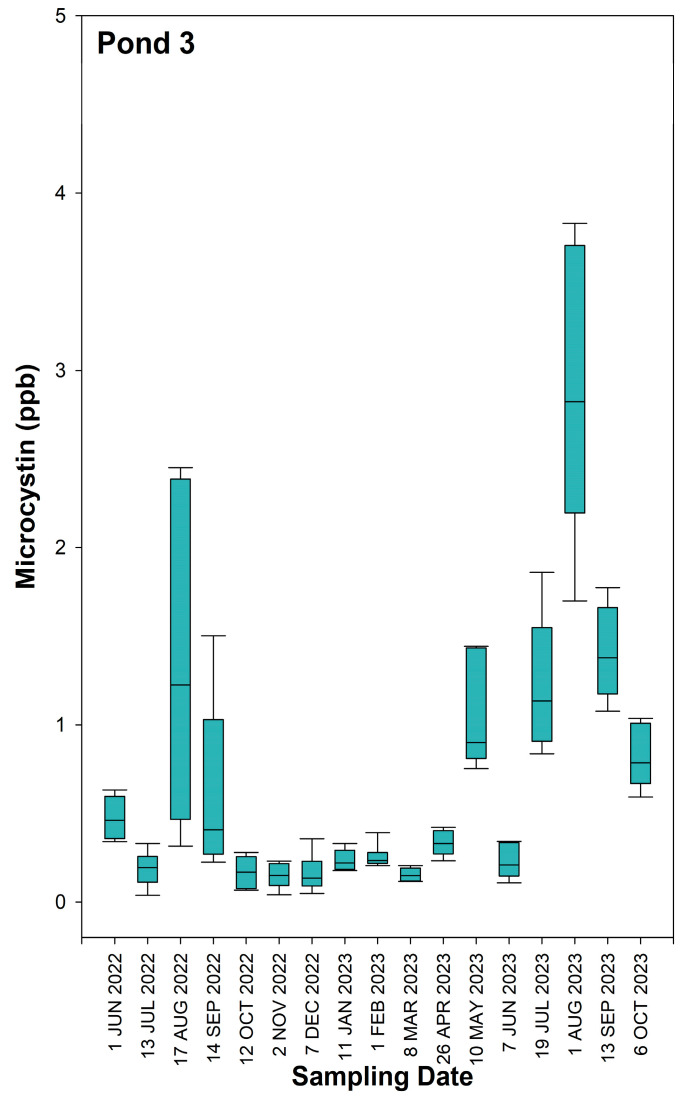
Box plots of microcystin concentrations in Pond 3 over the 17-month study period. Medians are displayed with solid lines. The upper and lower sides of the box represent the 1st and 3rd quartiles, with the distance between the quartiles being the interquartile range of microcystin concentration distributions for each sampling day. The whiskers show the minimum and maximum values that are not considered outliers.

The time series data of MC concentrations in Pond 1, Pond 2, and Pond 3 are represented in Figure 1, Figure 2 and Figure 3, respectively. Descriptive statistics for MC concentrations in each of the three ponds for individual sampling dates and for the combined 17-month study period are reported in Supplementary Table S1. Location maps showing the three locations with the highest MC concentrations for each individual sampling day at Pond 1, Pond 2, and Pond 3 can be found in Supplemental Tables S2 and S3, respectively. Among the three ponds, Pond 3 had the lowest concentrations of MC, with a 17-month average of 0.69 ppb, a maximum of 3.83 ppb, and a minimum of 0.04 ppb. Pond 1 had the highest recorded MC concentration of 743.75 ppb. However, the median and mean MC concentrations of 4.87 and 14.11 ppb, respectively, were slightly less than what was recorded for Pond 2. The maximum MC concentration recorded for Pond 2 was 589.00 ppb, and the median and mean values were 5.91 and 14.28 ppb, respectively.

In Pond 1, the highest median MC concentrations were observed on the sampling dates of 18 February 2023 and 15 March 2023, with concentrations of 19.02 and 22.54 ppb, respectively. The largest outliers (>200 ppb) were seen on the previously mentioned sampling dates in addition to 10 August 2022 and 27 July 2023. MC concentrations were lowest at Pond 1 from August of 2023 to October of 2023, with the median concentrations being <2 ppb. In Pond 2, the highest median concentrations of MC were observed on 19 July 2023 and 1 August 2023, with median concentrations of 12.42 and 15.56 ppb, respectively. The largest outlier (>200 ppb) for Pond 2 was seen on 7 December 2022, with a concentration of 589.00 ppb. The lowest median concentrations (<2 ppb) were observed in Pond 2 on 13 July 2022, 11 January 2023, and 1 February 2023. In Pond 3, all dates except for 1 August 2023 had median MC concentrations <2 ppb, and the largest outlier throughout the entire 17-month study period was 3.83 ppb.

#### 2.1.3. Water Quality Measurements

Descriptive statistics for the eight measured water quality parameters in each of the three ponds over the study period are reported in Supplemental Table S4. Pond 1 had the highest median and mean values for CHL, Phyco, and NTU when compared to the other ponds. Pond 2 had the highest median and mean values for DO, SPC, and pH. The median and mean TEMP and FDOM values were the highest in Pond 3. Phyco values varied drastically in Pond 1 (1.18–160.54 RFU) and Pond 2 (0.37–127.69 RFU) but not in Pond 3 (0.09–6.86 RFU). The CHL range was widest in Pond 1, and Pond 3 had higher CHL values than Pond 2 despite having the lowest Phyco values. Water temperature in all three ponds followed an expected seasonal trend, with lower values occurring in winter months compared to summer months. For Ponds 1, 2, and 3, respectively, the mean winter temperatures of the ponds were 15.68, 17.52, and 18.12, and the mean summer temperatures were 29.55, 29.51, and 30.29.

### 2.2. Spatial and Temporal Stability of Microcystin and Wind Data

The temporal stability of MC within pond waters was assessed by considering the standard errors of the mean relative differences (MRDs) for each location. The MRD graphs for each pond can be found in Supplemental Figure S3. Small standard errors indicate that a location had minimal MC concentration variations between each sampling date, and a large standard error indicates substantial variations in MC between sampling dates. Overall, locations that were consistently below a given pond’s daily average tended to be interior zones, and locations that were consistently higher than the pond’s daily average tended to be nearshore zones. Wind data were collected from weather stations within 1 km of all three ponds. Sampling locations with consistently higher and consistently lower concentrations of MC as they corresponding wind speed and directions depicted as wind roses are described in Section 2.2.1, Section 2.2.2 and Section 2.2.3. Wind vector plots showing the direction and magnitude of winds for each individual sampling day at Pond 1 and Pond 2 can be seen in Supplemental Tables S3 and S4, respectively.

#### 2.2.1. Pond 1 Mean Relative Differences and Wind Data

**Figure 4:**
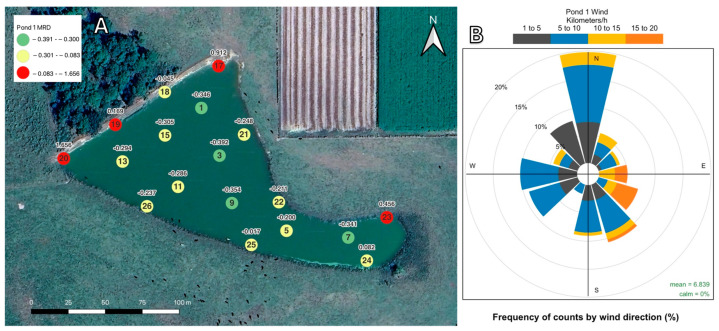
(**A**). The MRD of MC over the 17-month study period for Pond 1. The number inside each circle indicates the sampling location’s name, and the number above indicates the MRD value. Locations with MRD values below the 25th percentile are displayed in green, locations above the 75th percentile are displayed in red, and locations between the 25th and 75th percentile are displayed in yellow. (**B**). Wind speed and direction frequencies for Pond 1 are displayed in the insert. Wind speed is reported in km/h, and the direction corresponds to the direction the wind is blowing to.

Geographic representations of the MRD for each location at Pond 1 may be found in Figure 4A. The sampling locations at Pond 1 with consistently lower MC concentrations than the daily pond average over the 17-month study period were all interior zones. The zones with consistently higher MC concentrations were all nearshore sampling locations, with most of the higher concentration zones being located on the northern and northwestern banks. Sampling locations 17 and 23 are shallow and are where cattle are often seen entering and exiting the pond. Locations 19 and 20 are located near a dam that feeds a small creek. The wind data are visualized in Figure 4B. The most frequent wind direction at Pond 1 was northerly. The greatest wind speeds at Pond 1 were between 15 and 20 km/h and were associated with the east and southeast wind directions. These stronger wind events occurred approximately 5 to 10% of the time.

#### 2.2.2. Pond 2 Mean Relative Differences and Wind Data

**Figure 5:**
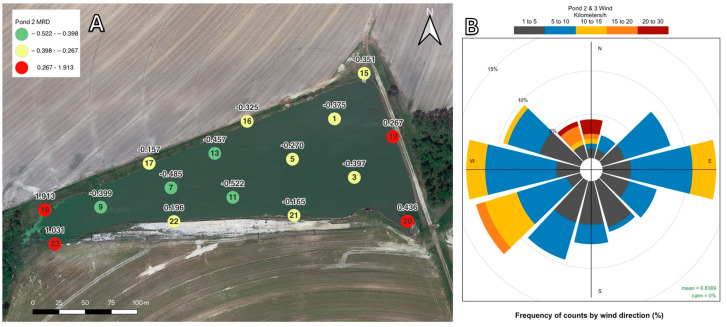
(**A**). The MRD of MC over the 17-month study period for Pond 2. The number inside each circle indicates the sampling location’s name, and the number above indicates the MRD value. Locations with MRD values below the 25th percentile are displayed in green, locations above the 75th percentile are displayed in red, and locations between the 25th and 75th percentiles are displayed in yellow. (**B**). Wind speed and direction frequencies for Pond 2 and Pond 3 are displayed in the insert. Wind speed is reported in km/h, and the direction corresponds to the direction the wind is blowing to.

Geographic representations of the MRD for each location at Pond 2 can be found in Figure 5A. As with Pond 1, the locations with MC concentrations that were consistently lower than the daily pond averages over the entire study period were all interior zones—specifically, the western interior locations of the pond. The locations with consistently higher concentrations of MC were all nearshore zones associated with the western and eastern banks. An irrigation intake pump is located along the bank between locations 15 and 16. Locations 18, 20, and 23 are associated with shallow water depths and submerged aquatic vegetation. The wind data are visualized in Figure 5B. The most frequent wind directions at Pond 2 were moving to the east, southwest, and west. The greatest wind speeds were between 20 to 30 km/h and were associated with the north and northwest directions.

#### 2.2.3. Pond 3 Mean Relative Differences and Wind Data

**Figure 6:**
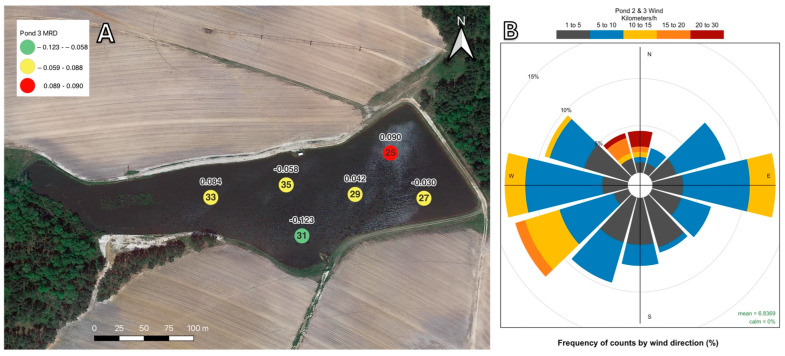
(**A**). The MRD of MC over the 17-month study period for Pond 3. The number inside each circle indicates the sampling location’s name, and the number above indicates the MRD value. Locations with MRD values below the 25th percentile are displayed in green, locations above the 75th percentile are displayed in red, and the locations between the 25th and 75th percentiles are displayed in yellow. (**B**). Wind speed and direction frequencies for Pond 2 and Pond 3 are displayed in the insert. Wind speed is reported in km/h, and the direction corresponds to the direction the wind is blowing to.

Geographic representations of the MRD for each location at Pond 3 can be found in Figure 6A. The location with consistently low MC concentrations compared to the pond’s daily averages over the study period was location 31, and the location with consistently high MC concentrations was location 25. All sampling locations at this pond are considered interior locations, and no nearshore samples were taken. An irrigation intake pump is located on the bank to the northeast of location 35, and there is a forested island to the west of location 33. The areas to the west of location 33 were too shallow to sample by boat. In general, the MRD value ranges were smaller than those observed in Pond 2 and Pond 1. The wind data are visualized in Figure 6B. The most frequent wind directions at Pond 3 were moving to the easter, southwest, and west. The greatest wind speeds were between 20 and 30 km/h and were associated with the north and northwest directions.

### 2.3. Spatial and Temporal Stability of Water Quality Parameters

**Figure 7:**
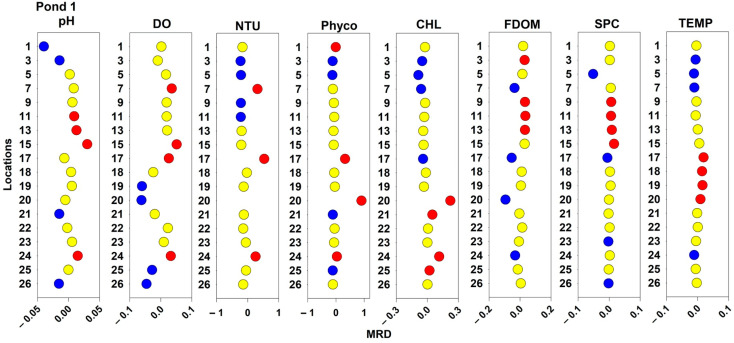
Spatial patterns of water quality parameters for Pond 1. Locations with MRD values below the 25th percentile are displayed in blue, locations above the 75th percentile are displayed in red, and locations between the 25th and 75th percentiles are displayed in yellow.

**Figure 8:**
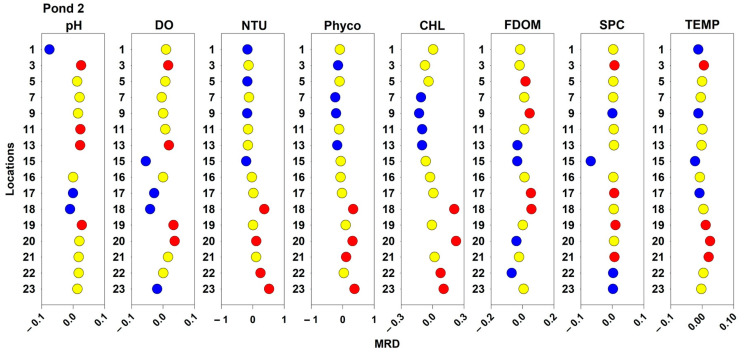
Spatial patterns of water quality parameters for Pond 2. Locations with MRD values below the 25th percentile are displayed in blue, locations above the 75th percentile are displayed in red, and the locations between the 25th and 75th percentiles are displayed in yellow.

**Figure 9:**
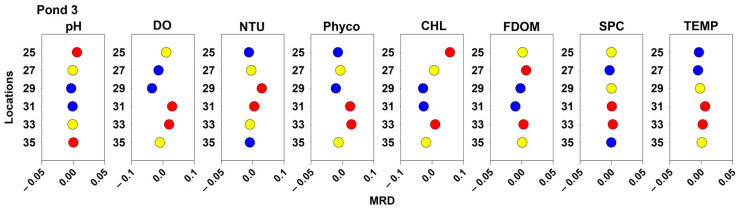
Spatial patterns of water quality parameters for Pond 3. Locations with MRD values below the 25th percentile are displayed in blue, locations above the 75th percentile are displayed in red, and locations between the 25th and 75th percentiles are displayed in yellow.

Spatial pattern MRDs for the measured water quality patterns are displayed in Figure 7, Figure 8 and Figure 9 for Ponds 1, 2, and 3, respectively. In Pond 1, measured values for sampling location 20 were consistently higher than the pond averages for NTU, Phyco, CHL, and TEMP but consistently below average for DO and FDOM. Additionally, at sampling location 17, DO, NTU, Phyco, and TEMP values were consistently above the daily averages. At the same sampling location, CHL values were consistently below the pond’s daily averages. For TEMP, all the locations along the northern bank (17–20) by the forested area had average higher water temperatures. For NTU in Pond 1, the sampling locations with consistently low turbidities were all interior locations; and for FDOM and SPC, all the consistently above-average locations were interior locations. In Pond 2, all sampling locations that were above the 75th percentile for NTU, Phyco, and CHL measurements were nearshore locations. Additionally, the sampling locations with consistently above-average water temperatures were in the southeast corner of the pond (3, 19, 20, and 21). Sampling location 15 in Pond 2 had consistently below-average values for pH, DO, NTU, FDOM, SPC, and TEMP. Interior sampling locations tended to have consistently below average NTU, CHL, and Phyco, and nearshore locations had consistently higher than average NTU, CHL, and Phyco values. Sampling location 20 in Pond 2 had consistently above average values for DO, NTU, Phyco, CHL, and TEMP. In Pond 3, location 31 had consistently above-average values for DO, NTU, Phyco, SPC, and TEMP. Additionally, location 33 had consistently above-average values for DO, NTU, Phyco, CHL, SPC, and TEMP. The sampling location at Pond 3 that had consistently low values for pH, DO, Phyco, CHL, and FDOM was location 29.

### 2.4. Microcystin and Water Quality Mean Relative Difference Correlations

The Spearman’s rank correlations between the mean relative differences of the water quality parameters and the mean relative differences of MC concentrations for all three ponds are displayed in Table 1. The critical r_s_ values based on the number of pairs and a significance value of 0.05 were determined to be 0.400, 0.426, and 0.729 for Pond 1, Pond 2, and Pond 3, respectively. Significant correlations between water quality MRDs and MC MRDs are bolded and italicized Table 1. MC MRDs showed significant positive correlation with chlorophyll a, phycocyanin, and turbidity in both Pond 1 and Pond 2. Additionally, in Pond 1, SPC and FDOM were significantly negatively correlated with MC concentrations. There were no significant correlations found between water quality MRDs and MC MRDs in Pond 3.

## 3. Discussion

Agricultural ponds serve numerous functions on working farms. Primarily, they are used for irrigation of crops and are often used by livestock as a drinking water source. In tropical and subtropical locations, these waters can also be used to help the animals cool off during the summer months [50,51]. These activities can cause health concerns when cyanobacteria and their toxins are present. Despite this, there are currently no MC thresholds or regulations for agricultural irrigation waters or livestock watering ponds. It is well established that MC can be transported to nearby crops and fields during irrigation and even be taken up through the roots of crops and produce [5,6,52]. Globally, toxicosis has been documented in varying species of livestock, including cattle [53,54], sheep [55], and swine [56]. Livestock exposure to cyanotoxins occurs through drinking and through wading/swimming behaviors wherein dense cyanobacterial scums stick to the coats of livestock; the cyanobacteria may then be ingested through grooming behaviors [33]. More recently, livestock exposure to MCs was documented to occur through feeding with alfalfa that had been irrigated with MC-laden waters [34]. Regionally, a study of 41 shallow livestock watering ponds in Alabama demonstrated that MC was present along the shorelines of all ponds to which livestock had direct access [57]. During this study, we witnessed livestock accessing the waters of Pond 1 via the bank where MC concentrations were the highest.

In Pond 1 and Pond 2, the sampling locations with the largest MC MRDs were entirely nearshore sampling locations where blooms of *Microcystis aeruginosa* and *M. wesenbergii* (Pond 1) and *M. aeruginosa*, *M. wesenbergii*, and *M. panniformis* (Pond 2) were noted throughout summer and winter sampling events [58]. For Pond 1 (Figure 4), MC concentrations were greatest along the north and northwest shorelines, corresponding both to the locations where *Microcystis* blooms were visible and to the prevailing wind directions during the study. Similarly, for Pond 2 (Figure 5), MC concentrations were greatest along the western and eastern shorelines, corresponding to the places where *Microcystis* blooms were visible and the prevailing wind directions during the study. This pattern of higher concentrations of MC near the shoreline compared to interior water has been reported in other studies on freshwater sources. There are several proposed factors leading to the higher MC concentrations at the shoreline. Chaffin et al. [59] and Palagama et al. [35] reported MC levels being higher at nearshore sampling locations than in offshore or interior waters in Lake Erie and attributed this to greater nutrient availability along the shoreline. Additionally, Palagama et al. [35] indicated that more MC congeners were found at nearshore locations in Lake Erie due to nutrient loading from the inflow of the Maumee River. Similar loading patterns may partially explain the reasons for Pond 2 locations 18 and 23 having the highest MRDs for MC. The inflow from a small creek that runs through a dairy cattle farm enters Pond 2 between locations 18 and 23 and could be acting as a point source of nutrients.

Several studies have indicated that *Microcystis* population buildup along a shoreline is directly related to wind direction and speed and that scums or blooms tend to form by horizontal shifts across the water due to wind [20,60,61]. In the case of Pond 1, the locations whose MC concentrations were consistently higher than the daily pond average were mostly sites along the northern bank and location 23. This corresponded to the frequency of northerly winds during and specifically three hours prior to sampling. At Pond 1, over the course of the study, approximately 34% of all winds were blowing in a northerly direction, facilitating the build-up of *Microcystis* populations and MC concentrations along the northern shoreline. When looking at individual sampling dates, wind direction can explain the three locations with the highest MC concentrations on 15 of 17 sampling dates. Similarly, at Pond 2, the locations that were consistently higher than the pond average were found on the eastern and western banks of the pond. These zones with consistently higher concentrations of MC may be explained by wind direction. At Pond 2, approximately 34% of the measured winds three hours prior to and during sampling were blowing to the northwest, west, and southwest, and approximately 28% of the winds were blowing to the northeast, east, and southeast. Since MC concentrations throughout Pond 3 were near the lower limit of detection during this study, correlations between MC concentration and locations within the pond cannot be established. However, the data collected at Pond 3 during this study can form a baseline assessment for future investigations at this location.

The spatial patterns of MC correlated well with certain water quality patterns in Pond 1 and Pond 2. Pond 3 showed no significant correlations between spatial water quality patterns and spatial MC patterns. Only a few studies have reported on the correlations between patterns rather than measured values themselves. The MRDs of MC were significantly and positively correlated with the spatial patterns of chlorophyll a, phycocyanin, and turbidity for both Pond 1 and Pond 2. Singh et al. [62] used principal component analysis (PCA) to validate that the spatial patterns of MC concentrations had significant positive correlations with the spatial patterns of chlorophyll a, biomass, nutrient levels, and rainfall. Additionally, in a Vietnamese reservoir, it was demonstrated using PCA that MC concentration patterns correlated with water temperature and phosphate [63]. Numerous studies have documented that MC values may be correlated with values of water quality variables such as chlorophyll a [49,62,64], phycocyanin [65], turbidity [66], water temperature [67], nutrients [68,69], biovolume [70,71], carbon [72], and pH [69]. If correlations between water quality measurements and MC can be established, these variables may provide insights for MC monitoring practices. The examination of MCs in water samples via the ELISA method or other available techniques is an expensive procedure requiring both a laboratory setup and trained personnel to analyze samples and interpret results. If strong correlations can be determined for water quality and MC concentrations, the possibility of using near-instantaneous in situ measurements in lieu of traditional toxin analyses could be further investigated to augment resource monitoring and management practices.

While *Microcystis* blooms and their corresponding MC toxin concentrations are commonly reported at the warm water temperatures that occur in summer (summarized in Wood et al. [73]), fewer reports are available on *Microcystis* blooms and MC toxin production in winter. Studies that monitor MC concentrations throughout the winter months often report low or undetectable concentrations of MC during these times [74,75,76,77]. Most MC surveys report detectable concentrations from early summer to late fall, with peak MC levels recorded in the late summer and early fall months [22,62,78]. Within Georgia, HAB monitoring is only conducted bi-weekly in the summer months [79]; therefore, winter *Microcystis* blooms and their resulting toxins would not be captured by this routine surveillance program. In two of the agricultural ponds we examined in Georgia, both *Microcystis* and MC were detected over the winter period. For both ponds, the highest mean MC concentrations recorded during the 17-month survey were detected in winter months: February 2023 (65.58 ppb) and December 2022 (52.45 ppb) in Pond 1 and Pond 2, respectively.

Two scenarios may be contributing to elevated cell and toxin concentrations in winter. In these shallow ponds, meteorological events may be supporting benthic *Microcystis* populations. Ma et al. [80] found that in shallow water bodies, sunny winter days can warm waters enough and provide enough light to the benthos to stimulate the growth of *Microcystis*, even when water temperatures are initially around 10 °C. This process can be accelerated if there is a *Microcystis* population overwintering in the water column [81]. Our work in Georgia indicated that for all of the winter months, water temperatures were well above this potentially growth-inhibitory threshold of 10 °C, indicating that *Microcystis* may have been growing throughout the winter in these waters. Weber et al. [31] also highlighted the importance of winter temperatures in sustaining cyanoHABs in the Georgia Piedmont region. Sampling these ponds on a more frequent basis and/or conducting temperature–growth rate experiments with local strains would help to address the sensitivity of local *Microcystis* strains to various temperature ranges.

It has also been determined that wind plays a significant factor in supporting *Microcystis* blooms, particularly in shallow waters. Tammeorg et al. [82] found that wind not only resuspends *Microcystis* into the water column but also releases nutrients from the benthos that are readily available for usage by cyanobacteria. Similarly, Wu et al. [83] reported that *Microcystis* blooms can alter the surface tension of water as a competitive advantage over other phytoplankton species; in turn, this allows *Microcystis* to have an increased rate of surface bloom re-formation and lateral expansion after strong wind events. In the Southeastern US, the fewest high-wind events (either sustained or gusts) occur in the winter [84], providing ample time for a stable water column to be established and dense surface blooms of *Microcystis* to form.

The second factor that may be contributing to elevated MC concentrations in the winter is changes to the microbial heterotrophic community. Li et al. [85] and Lezcano et al. [86] showed that both nutrient concentrations and bacterial community structure within biofilms impacted the speed at which MC was broken down. Over winter, MC degradation efficiency was reduced as the bacteria community shifted to more cold-tolerant species and the concentrations of MC-degrading bacteria were lessened [85]. Similarly, Chen et al. [87] and Zhang et al. [88] have shown that shifts in the heterotrophic algae community can also reduce the biodegradation of MCs at temperatures below 15–20 °C, the same temperature range at which *Microcystis* cells start to release more extracellular MC. Reduced biodegradation capacity coupled with an increase in extracellular MC output may explain the elevated MC concentrations we detected during winter sampling. Examination of the phytoplankton community structure in these ponds is ongoing, including a characterization of the heterotrophic algal community.

Overall, the spatiotemporal variation of MC concentrations is a complex phenomenon. The importance of its monitoring indicates the need to understand further the effect of multiple factors that create wide MC variation even in relatively small water bodies. Sampling locations and frequency need to be tied to the use of water (e.g., recreation, irrigation, and animal husbandry), local conditions for physical MC concentration (e.g., water stagnancy and wind direction), and sources of organic matter and nutrients in the water (e.g., manure erosion, direct excreta deposition, and eutrophication processes). It remains to be seen at which taxonomic levels cyanobacteria should be characterized to anticipate MC concentration dynamics, as speciation has been deemed critical by Lezcano et al. [81], Bukowska et al. [89], and Wejnerowski et al. [90] and may be shifting with climate change pressures [73,91]. Farm ponds are relatively under-researched water bodies, and their functioning as year-round MC reservoirs demonstrates a need for additional research efforts.

## 4. Materials and Methods

### 4.1. Sites; Field and Laboratory Analyses

**Figure 10:**
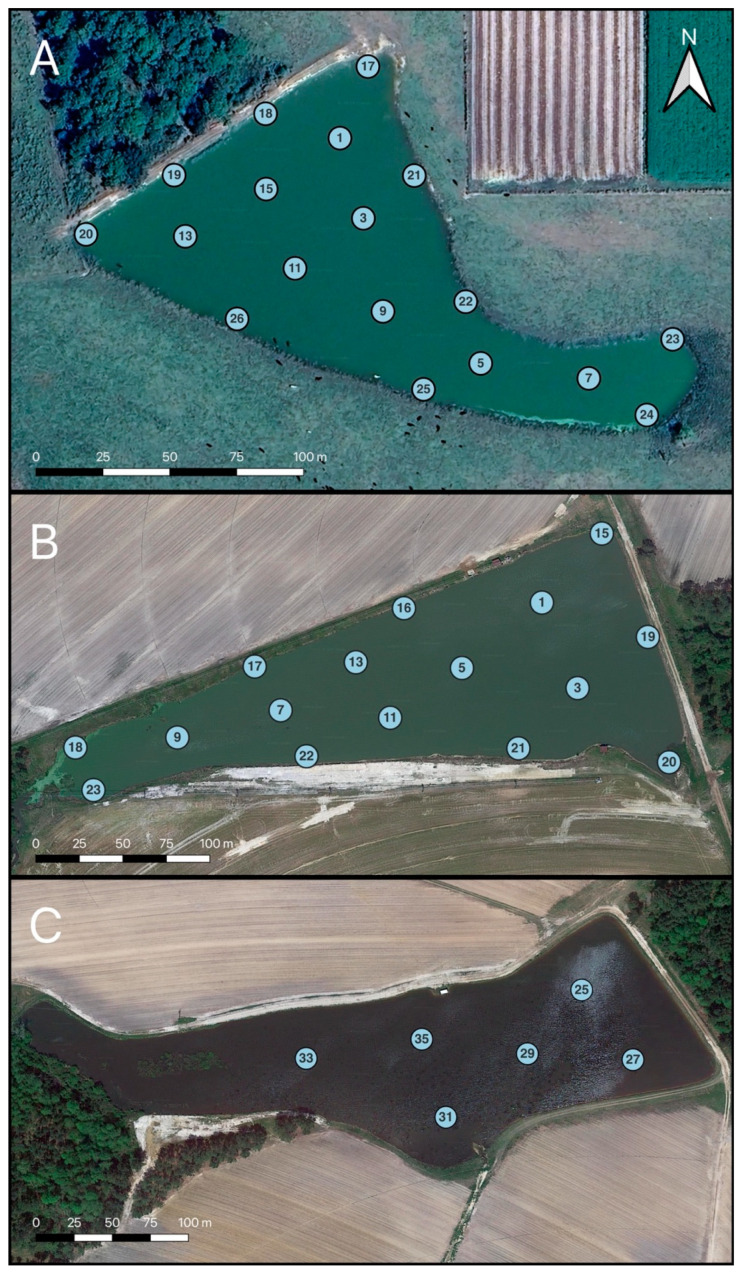
Sampling locations for Pond 1 (**A**), Pond 2 (**B**), and Pond 3 (**C**).

Water sampling was performed at three agricultural ponds on two farms in South Georgia, USA. These agricultural ponds are referred to as Pond 1 (Figure 10A), Pond 2 (Figure 10B), and Pond 3 (Figure 10C). The names of the farms and ponds were replaced for anonymity. Pond 1 is approximately 16,000 m^2^, with an average depth of 1.14 m, and is located in Sumner, GA. The pond is currently utilized as an irrigation and livestock watering pond for a herd of approximately 50 beef cattle and is surrounded by pasture for the livestock. Pond 1 contained 18 fixed sampling locations resulting in the collection of 306 samples over the course of the study. Pond 2 and Pond 3 are located on the same farm in Ty Ty, GA, and are approximately 500 m apart from each other. Both ponds are used for irrigation of surrounding crops (rotation of corn and cotton, with no cover crops). Pond 2 has an area of approximately 32,000 m^2^, with an average depth of 1.17 m, and Pond 3 has an area of approximately 40,000 m^2^, with an average depth of 1.22 m. There were 16 and 6 fixed sampling locations, resulting in the collection of 272 and 102 samples over the course of the study, for Pond 2 and Pond 3, respectively. The land surrounding all three ponds is characterized by extensive sandy soils, with little (≤5% grade) to no slope (A. Yakirevich, pers. comm.). In Georgia, tilled loamy sand soils often experience water gains rather than overland or lateral losses during rain events, particularly in January–March and June–August [92].

Sampling of the ponds occurred monthly from June 2022 through October 2023 for a total of 17 sampling dates for each pond. Surface water grab samples (500 mL) were collected along a fixed sampling grid consisting of interior and nearshore zones. An Arrow Lite GPS (EOS, Terrebonne, QC, Canada) with sub-meter accuracy was used to provide consistency of sampling locations between different sampling dates. The interior zones were sampled by boat, and the nearshore samples were collected with a grab sampler from the shoreline. Samples were typically collected between 9 a.m. and 12 p.m. and were transported back to the laboratory where processing occurred on the same day. The study accumulated a combined total of 687 surface water samples for all three ponds. Water quality parameters were measured in situ with a YSI EXO-2 sonde (Yellow Springs Instruments, Yellow Springs, OH, USA) in the same locations where the water samples were taken. The sonde measured a total of eight parameters, including temperature (TEMP; °C), pH, specific conductivity (SPC; μS cm^−1^), fluorescent dissolved organic matter (FDOM; relative fluorescent units [RFU]), dissolved oxygen (DO; mg L^−1^), chlorophyll a (CHL; RFU), phycocyanin (Phyco; RFU), and turbidity (NTU; nephelometric turbidity units [NTU]). All measurements were conducted according to manufacturer guidelines.

The water samples were transported back to the laboratory, where 20 mL aliquots were taken from each 500 mL bottle after inverting them 25 times; the aliquots were frozen at −20 °C until analysis. MC toxin analysis was conducted using microcystin–ADDA ELISA kits (PN#520011) from Eurofins Abraxis (now Gold Standard Diagnostics; Warminster, PA, USA) after each sample went through three freeze–thaw cycles. Either the sample dilutions were made prior to analysis or the samples were rerun with proper dilutions to fit within the ELISA kit’s standard curve. Each ELISA measurement was performed in duplicate and analyzed on a microplate reader according to manufacturer recommendations (Eurofins Abraxis, now Gold Standard Diagnostics; Warminster, PA, USA) according to the kit instructions.

Field and laboratory research safety guidelines followed the recommendations of the USDA’s environmental field and safety plan [93] and the University of Georgia’s health and safety management system [94].

### 4.2. Weather Conditions

Corresponding weather data for Pond 1 were retrieved from a weather station located on the property 200 m from the pond. Corresponding weather data for Ponds 2 and 3 were retrieved from a weather station located on the property 2.5 km from both ponds. Rainfall input was measured hourly from both weather stations. Wind data were collected during the time of sampling and the three hours prior to sampling. Daily average air temperature data and precipitation data were collected for the entire 17-month study period.

### 4.3. Software and Statistics

To assess temporal and spatial patterns of MC within the ponds, the mean relative difference (MRD) method was utilized. MRD was used to indicate how an individual location compared to the pond average over multiple sampling dates and could reveal areas that were consistently higher or lower than the average for each measured parameter. The methods used for MRD analysis here follow the methods described in previous spatial pattern studies [47,95,96], wherein the relative difference RD*_ij_* between the observation of variable x at location *i* at time *j* (*x_ij_*) and the spatial average of x at the same time (〈*x*〉*_j_*) is defined as follows:

$${RD}_{ij}=\frac{x_{ij}-{\langle x\rangle }_{j}}{{\langle x\rangle }_{j}}$$

The MRD for location *i* then becomes

$${MRD}_{i}=\frac{1}{N_{t}}\sum_{j=1}^{j=N_{t}}{RD}_{ij}$$

where N*_t_* is the number of sampling days and *i* = 1, 2, …, N*_i_*, where N*_i_* is the total number of locations.

All statistical calculations were performed in PAST software v4.16 [97]. Location site maps were created using QGIS v3.22 and Google satellite map services in QGIS. All figures were created using Sigmaplot v13 (Systat Software, San Jose, CA, USA).

## Figures and Tables

**Table 1:** Spearman rank correlations between microcystin MRDs and water quality MRDs.

	Microcystin		
Water Quality Parameters	Pond 1 R Crit = 0.400	Pond 2 R Crit = 0.426	Pond 3 R Crit = 0.729
CHL	***0.412***	***0.903***	0.657
FDOM	***−0.699***	0.001	0.486
DO	−0.296	−0.115	−0.143
SPC	***−0.575***	0.182	0.143
Phyco	***0.478***	***0.926***	−0.257
NTU	***0.697***	***0.750***	−0.429
pH	−0.044	−0.265	0.314
TEMP	0.344	0.400	−0.429

Significant (*p* < 0.05) correlations are indicated with bold and italic font.

## Data Availability

The datasets generated and analyzed during the current study are not publicly available as of the submission date. The material is part of ongoing degree programs of J.E.S. and J.A.W. Data will become available after completing the degree programs and can be requested from the corresponding author, Y.P.

## Supplementary Materials

The following supporting information can be downloaded at: https://www.mdpi.com/article/10.3390/toxins16110482/s1, Supplemental Figure S1: Average daily air temperature and daily precipitation for Pond 1; Supplemental Figure S2: Average daily air temperature and daily precipitation for Ponds 2 and 3; Supplemental Figure S3: Mean relative differences for microcystin concentrations in Ponds 1, 2, and 3; Supplemental Table S1: Microcystin concentration (ppb) descriptive statistics for Pond 1, Pond 2, and Pond 3 for individual dates and entire 17-month study period; Supplemental Table S2: Wind speeds for each respective sampling date and three highest microcystin concentration locations for each date at Pond 1; Supplemental Table S3: Wind speeds for each respective sampling date and three highest microcystin concentration locations for each date at Pond 2. Supplementary Table S4: Descriptive statistics for measured water quality parameters of Pond 1, Pond 2, and Pond 3 for the 17-month study period.
