# Supplementary material for: Persistence of Microcystin in Three Agricultural Ponds in Georgia, USA
Source: Toxins (Basel). 2024 Nov 7;16(11):482. doi: 10.3390/toxins16110482 (PMC11598104; doi:10.3390/toxins16110482)
Supplement: Supplementary file 1 [file toxins-16-00482-s001.zip › toxins-3243070-supplementary.pdf]

Supplementary Materials: Persistence of Microcystin in Three Agricultural Ponds in Georgia, USA

Jaclyn E. Smith, James A. Widmer, Jennifer L. Wolny, Laurel L. Dunn, Matthew D. Stocker, Robert L. Hill, Oliva Pisani, Alisa W. Coffin and Yakov Pachepsky

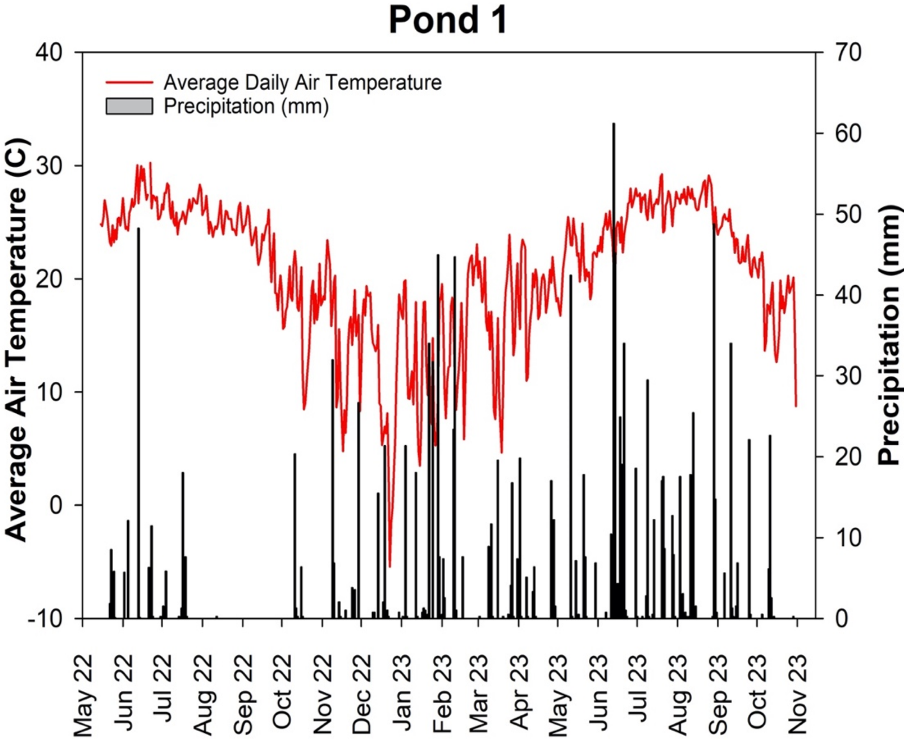

Figure S1. Average daily air temperature and daily precipitation for Pond 1.

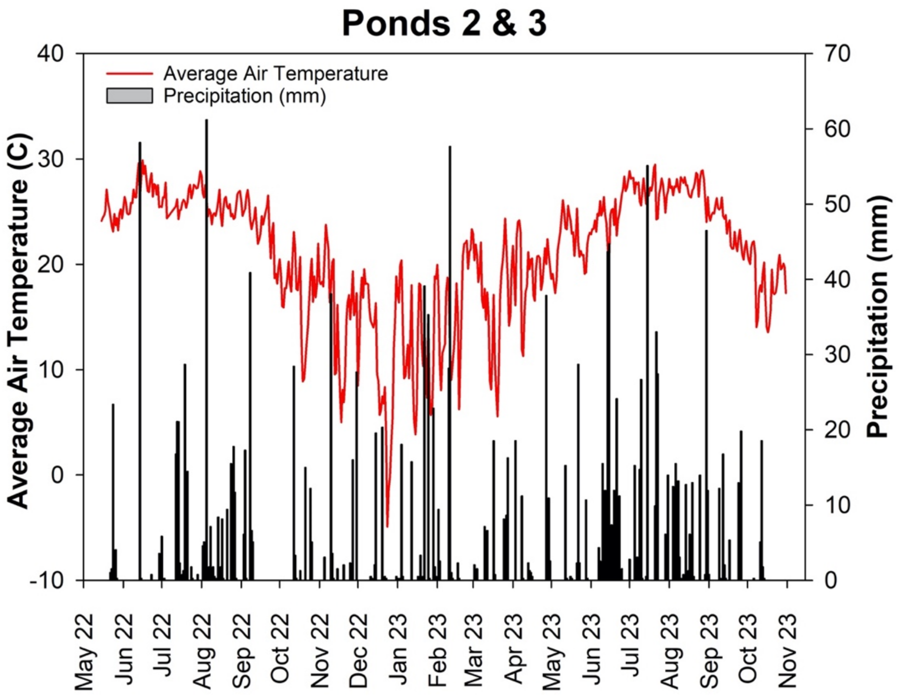

Figure S2. Average daily air temperature and daily precipitation for Ponds 2 and 3.

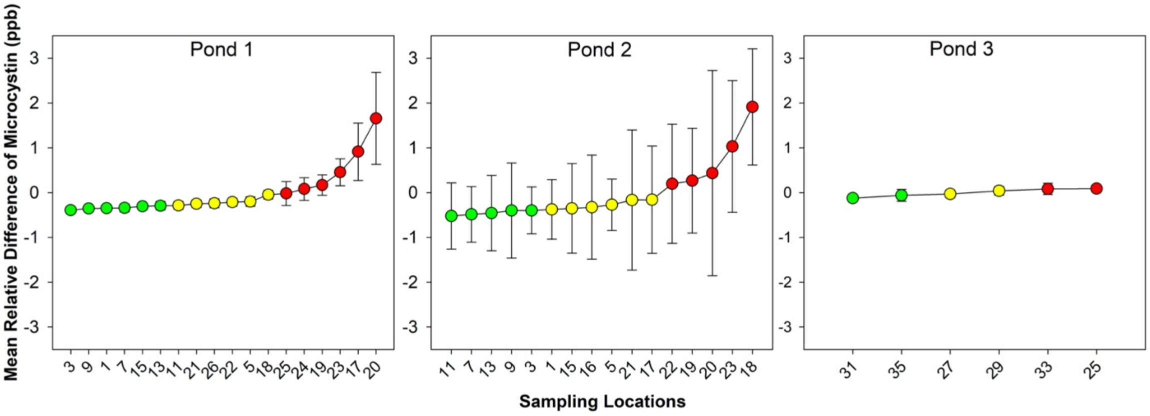

Figure S3. Mean relative differences for microcystin concentrations in Ponds 1, 2, and 3.

Table S1. Microcystin concentration (ppb) descriptive statistics for Pond 1, Pond 2, and Pond 3 for individual dates and entire 17-month study period.

Pond 1	Min	Max	Med	Mean
**All Dates**	**0.74**	**743.75**	**4.87**	**14.11**
09 JUN 2022	2.31	16.33	6.37	7.71
06 JUL 2022	2.74	31.48	4.13	6.43
10 AUG 2022	3.05	271.33	4.05	20.57
07 SEP 2022	1.71	12.97	2.61	3.40
05 OCT 2022	3.59	70.60	10.01	15.47
16 NOV 2022	5.47	13.49	10.14	10.23
14 DEC 2022	6.63	14.95	9.63	9.71
18 JAN 2023	8.42	83.80	10.44	18.54
08 FEB 2023	9.30	743.75	19.02	65.58
15 MAR 2023	1.97	143.10	22.54	28.70
05 MAY 2023	1.19	12.24	3.32	3.64
17 MAY 2023	1.99	4.66	3.20	3.26
20 JUN 2023	3.05	23.75	6.97	7.85
27 JUL 2023	2.01	275.58	4.56	19.75
09 AUG 2023	1.20	3.39	1.94	2.00
08 SEP 2023	0.74	2.50	1.12	1.26
18 OCT 2023	1.29	9.06	1.93	2.31
**Pond 2**	**Min**	**Max**	**Med**	**Mean**
**All Dates**	**0.50**	**589.00**	**5.91**	**14.28**
01 JUN 2022	2.25	84.63	3.04	11.44
13 JUL 2022	0.63	2.47	0.95	1.19
17 AUG 2022	1.53	32.01	3.67	6.65
14 SEP 2022	6.89	117.40	9.26	16.36
12 OCT 2022	2.77	154.78	4.61	13.88
02 NOV 2022	5.16	185.20	7.31	19.43
07 DEC 2022	1.96	589.00	4.12	52.45
11 JAN 2023	0.92	302.25	1.69	26.49
01 FEB 2023	0.50	5.91	0.84	1.50
08 MAR 2023	1.61	7.31	3.04	3.18
26 APR 2023	4.26	12.03	5.74	6.13
10 MAY 2023	6.18	16.60	9.44	9.98
07 JUN 2023	2.87	19.07	4.36	5.23
19 JUL 2023	8.42	145.50	12.42	20.66
01 AUG 2023	6.16	192.10	15.56	27.80
13 SEP 2023	5.23	10.91	7.09	7.91
06 OCT 2023	6.71	37.05	9.35	12.21
**Pond 3**	**Min**	**Max**	**Med**	**Mean**
**All Dates**	**0.04**	**3.83**	**0.33**	**0.69**
01 JUN 2022	0.34	0.63	0.46	0.47
13 JUL 2022	0.04	0.33	0.19	0.19
17 AUG 2022	0.32	2.45	1.22	1.35
14 SEP 2022	0.22	1.50	0.41	0.60
12 OCT 2022	0.07	0.28	0.17	0.17
02 NOV 2022	0.04	0.23	0.15	0.15
07 DEC 2022	0.05	0.36	0.13	0.16
11 JAN 2023	0.18	0.33	0.22	0.24

01 FEB 2023	0.21	0.39	0.23	0.26
08 MAR 2023	0.12	0.21	0.15	0.15
26 APR 2023	0.23	0.42	0.33	0.33
10 MAY 2023	0.75	1.44	0.90	1.04
07 JUN 2023	0.11	0.34	0.21	0.23
19 JUL 2023	0.84	1.86	1.13	1.22
01 AUG 2023	1.70	3.83	2.83	2.87
13 SEP 2023	1.08	1.77	1.38	1.41
06 OCT 2023	0.59	1.04	0.79	0.82

Table S2. Wind speeds for each respective sampling date and three highest microcystin concentration locations for each date at Pond 1.

<table>
<tr><th rowspan="2">Date</th><th rowspan="2">Wind Speed + Direction</th><th colspan="3">3 Highest Microcystin Concentration Locations</th></tr>
<tr><th>Maps</th><th>Loc</th><th>Conc (ppb)</th></tr>
<tr><td>09 JUN 2022</td><td>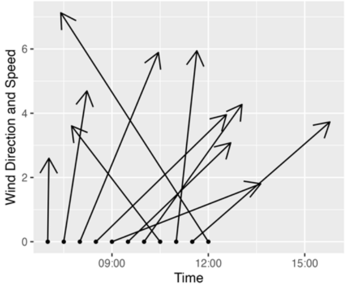
</td><td>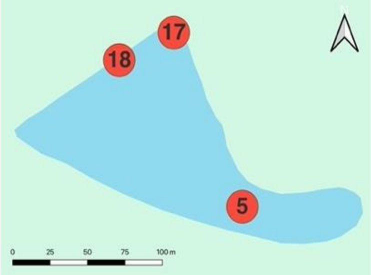
</td><td>18
17
5</td><td>16.325
15.425
13.930</td></tr>
<tr><td>06 JUL 2022</td><td>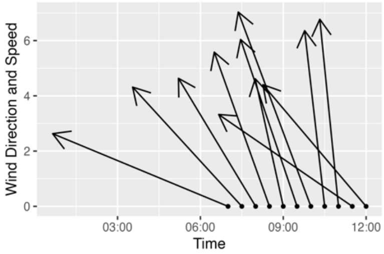
</td><td>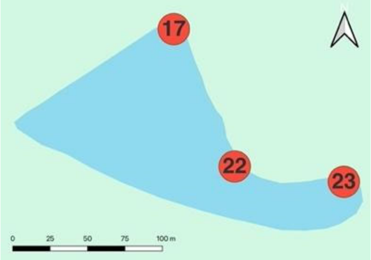
</td><td>23
17
22</td><td>31.475
15.855
9.555</td></tr>
<tr><td>10 AUG 2022</td><td>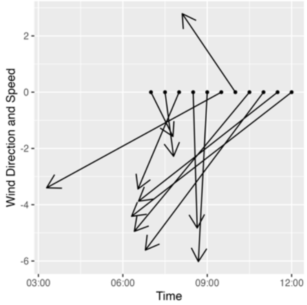
</td><td>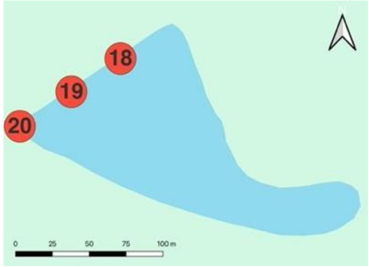
</td><td>20
19
18</td><td>271.325
21.030
16.425</td></tr>
<tr><td>07 SEP 2022</td><td>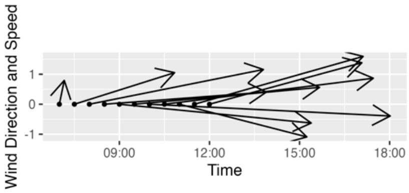
</td><td>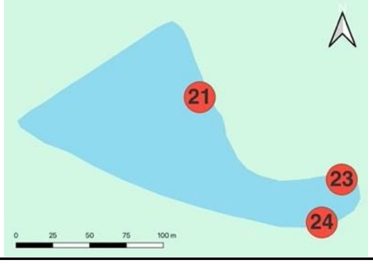
</td><td>23
24
21</td><td>12.973
7.210
3.369</td></tr>
</table>

05 OCT 2022	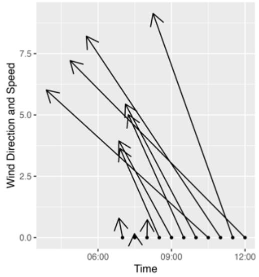	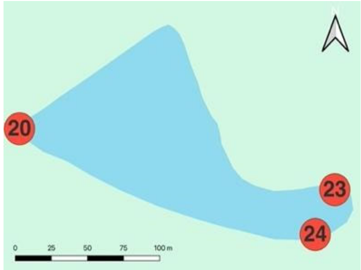	24<br>23<br>20	70.600<br>26.175<br>22.725
16 NOV 2022	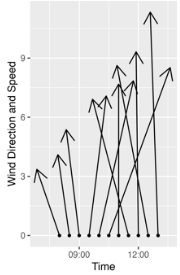	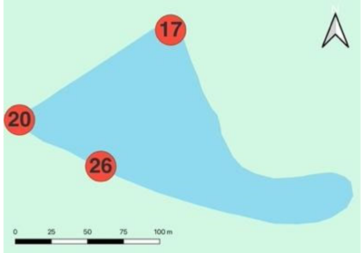	20<br>17<br>26	13.493<br>13.443<br>11.790
14 DEC 2022	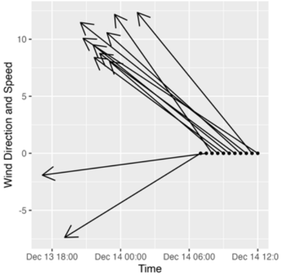	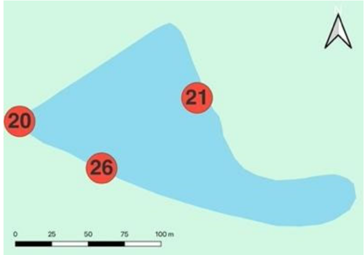	26<br>21<br>20	14.950<br>14.563<br>11.048
18 JAN 2023	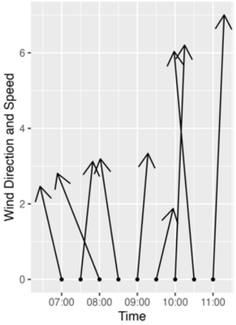	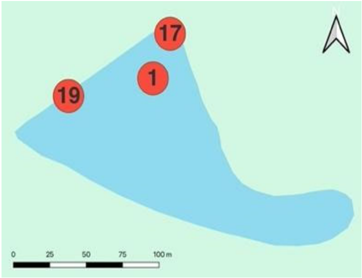	19<br>17<br>1	83.800<br>73.375<br>16.990
08 FEB 2023	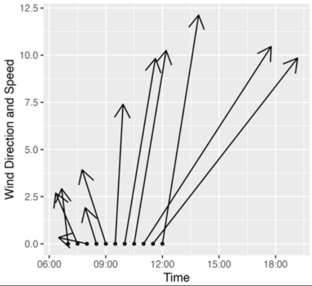	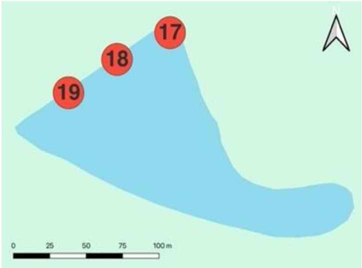	17<br>18<br>19	743.75<br>75.000<br>71.575

15 MAR 2023	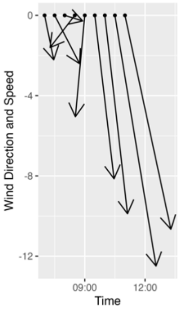	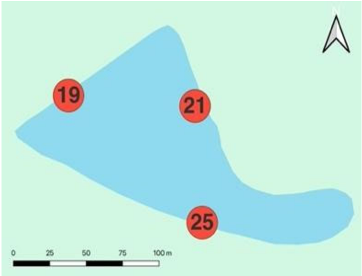	25<br>19<br>21	143.100<br>33.023 31.763
05 MAY 2023	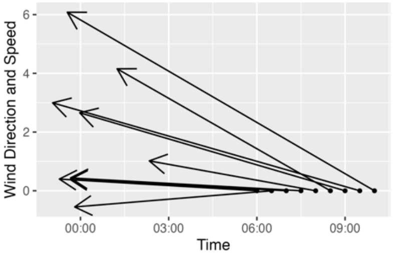	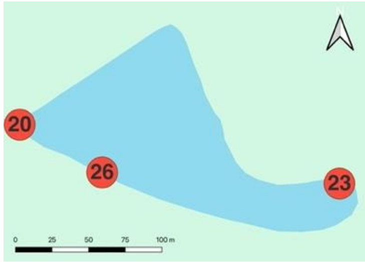	20<br>23<br>26	12.240<br>4.833<br>4.588
17 MAY 2023	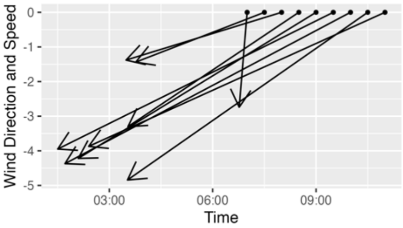	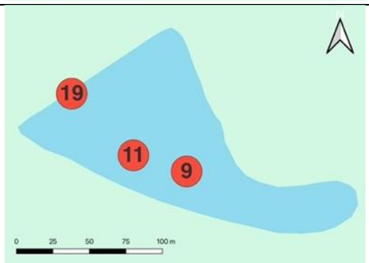	19<br>11<br>9	4.663<br>4.589<br>4.235
20 JUN 2023	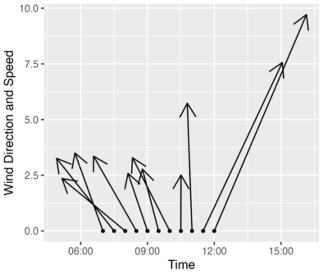	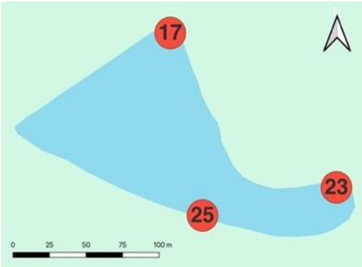	17<br>23<br>25	23.743<br>18.968<br>9.433
27 JUL 2023	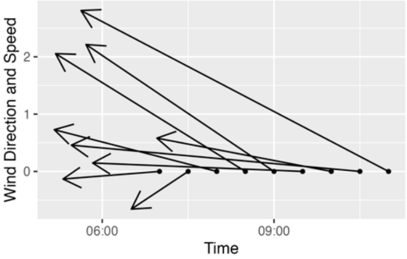	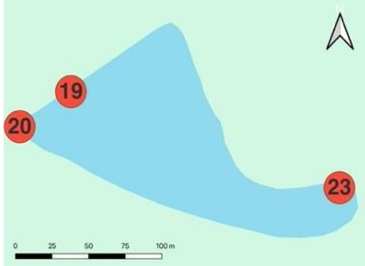	20<br>19<br>23	275.575<br>9.040<br>8.015
09 AUG 2023	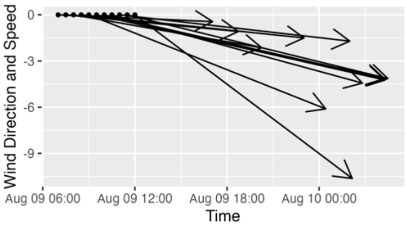	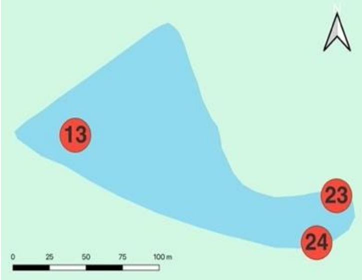	24<br>23<br>13	3.385<br>2.594<br>2.588

08 SEP 2023	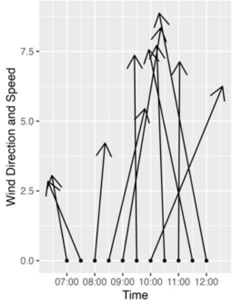	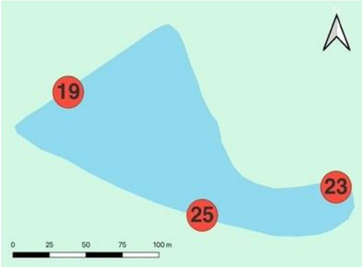	23<br>25<br>19	2.500<br>2.100<br>2.075
18 OCT 2023	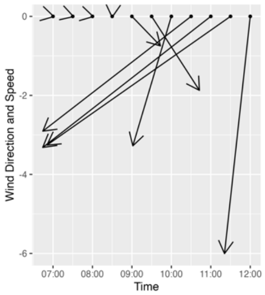	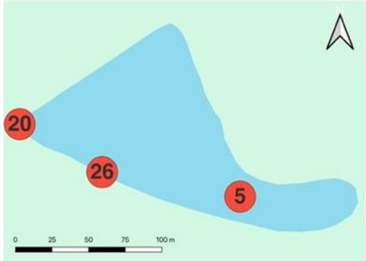	20<br>26<br>5	9.060<br>2.597<br>2.419

Table S3. Wind speeds for each respective sampling date and three highest microcystin concentration locations for each date at Pond 2.

**Date**	Wind Speed + Direction	3 Highest Microcystin Concentration Locations		
		Maps	Loc	Conc (ppb)
**01 JUN 2022**	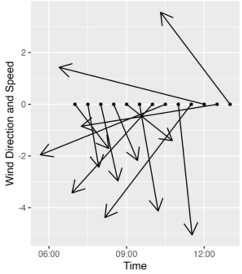	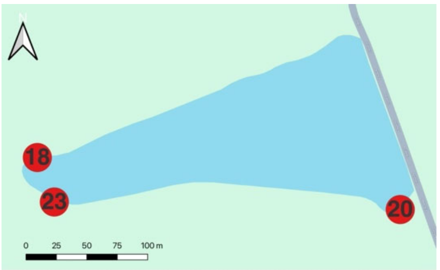	20<br>18<br>23	84.625<br>38.098<br>15.760
13 JUL 2022	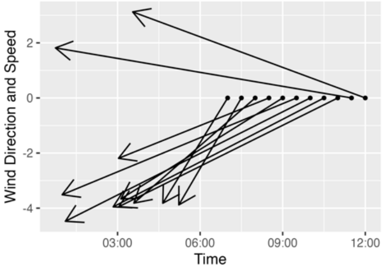	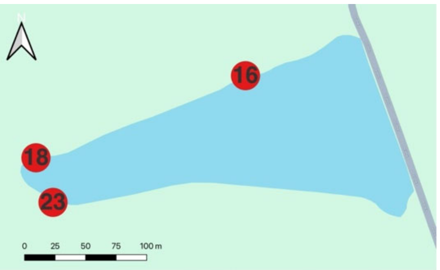	18<br>16<br>23	2.474<br>2.039<br>1.823
17 AUG 2022	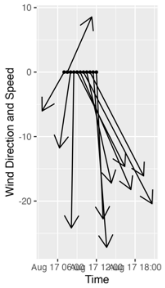	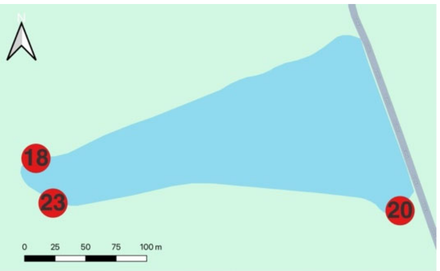	23<br>18<br>20	32.013<br>24.993<br>6.225

14 SEP 2022	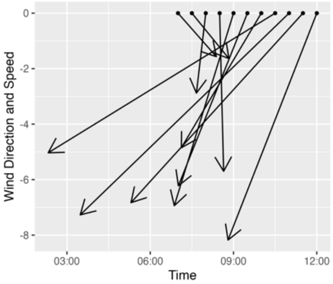	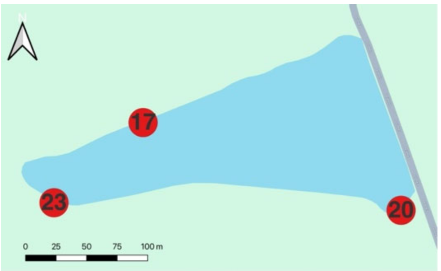	23<br>20<br>17	117.400<br>15.988<br>11.03
12 OCT 2022	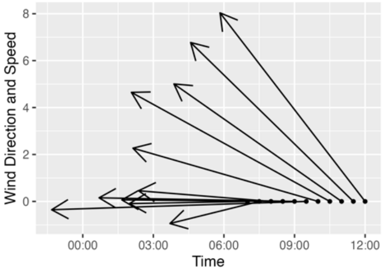	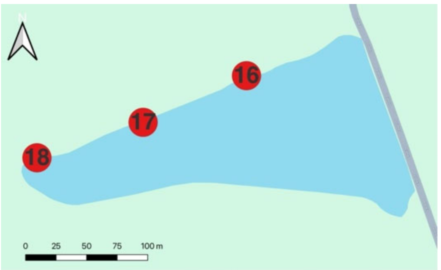	18<br>17<br>16	154.775<br>6.120<br>5.555
02 NOV 2022	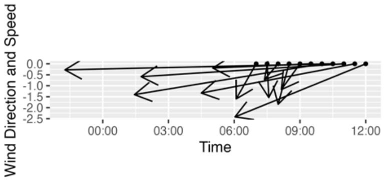	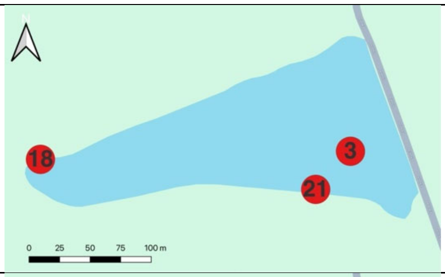	18<br>3<br>21	185.200<br>19.975<br>12.088
07 DEC 2022	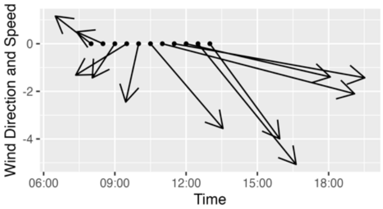	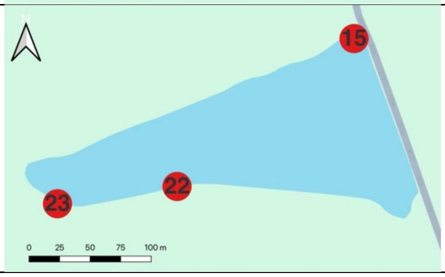	23<br>22<br>15	589.000<br>149.475<br>26.01
11 JAN 2023	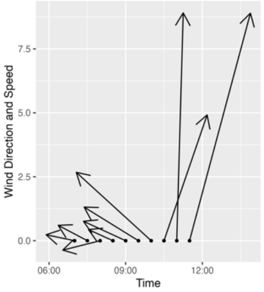	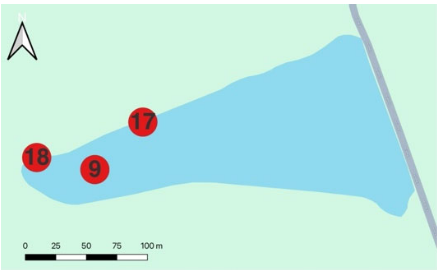	18<br>17<br>9	302.25<br>90.235<br>4.012
01 FEB 2023	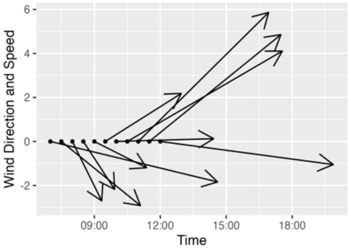	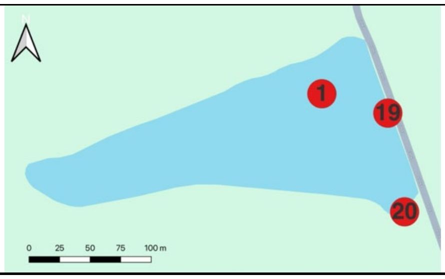	19<br>20<br>1	5.910<br>4.443<br>2.939

08 MAR 2023	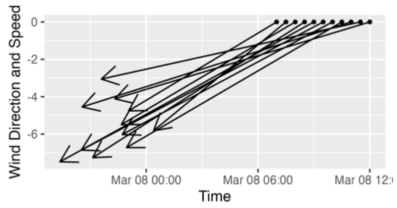	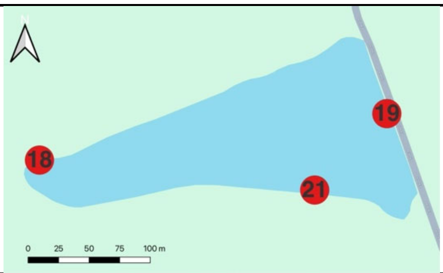	21<br>18<br>19	7.308<br>4.305<br>3.799
26 APR 2023	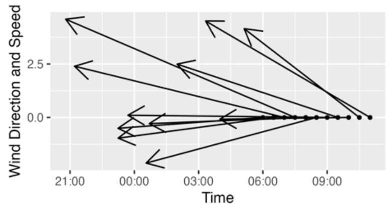	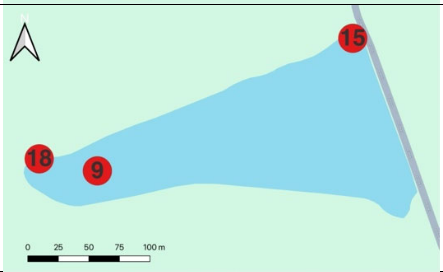	18<br>9<br>15	12.028<br>8.295<br>6.945
10 MAY 2023	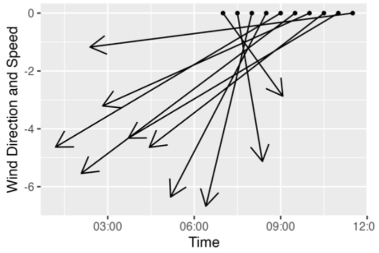	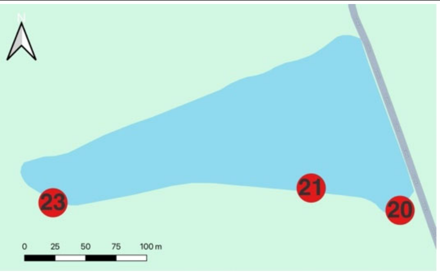	20<br>23<br>21	16.598<br>13.933<br>13.318
07 JUN 2023	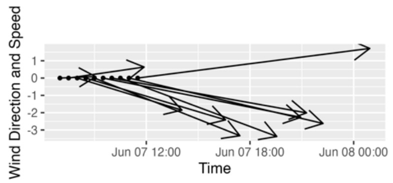	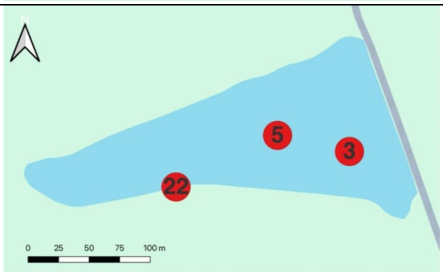	5<br>22<br>3	19.068<br>5.993<br>5.863
20 JUL 2023	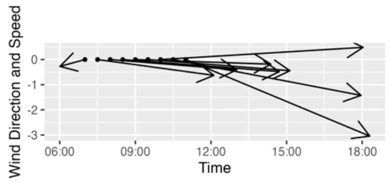	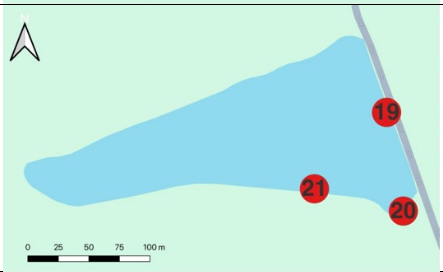	19<br>20<br>21	145.500<br>17.263<br>16.96
01 AUG 2023	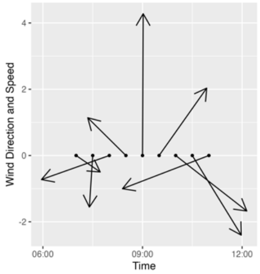	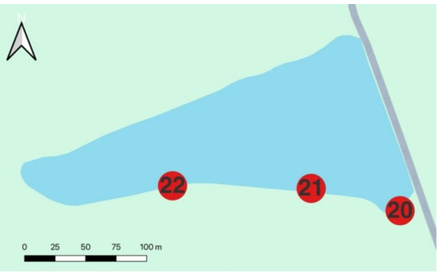	22<br>20<br>21	192.100<br>35.065<br>26.160

13 SEP 2023	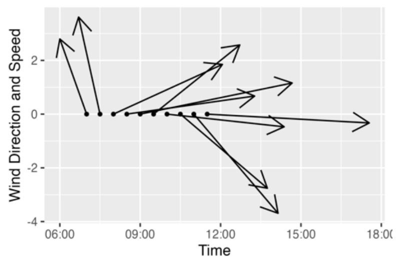	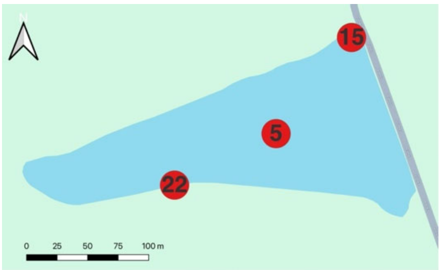	22<br>15<br>5	10.908<br>10.868<br>10.598
06 OCT 2023	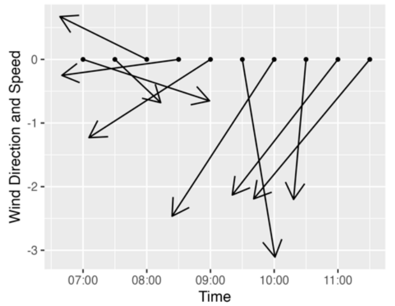	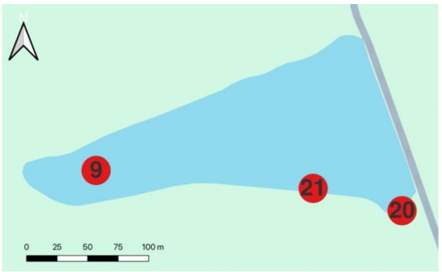	20<br>21<br>9	37.050<br>19.458<br>16.623

Table S4. Descriptive statistics for measured water quality parameters of Pond 1, Pond 2, and Pond 3 for the 17-month study period.

**Pond 1**	**Min**	**Max**	**Med**	**Mean**
CHL	0.94	40.95	8.68	11.97
FDOM	1.03	22.44	16.50	16.51
DO	2.96	13.95	9.59	9.42
SPC	7.10	151.50	120.30	124.60
Phyco	1.18	160.54	8.95	10.01
NTU	3.70	2657.98	55.94	79.79
pH	5.49	9.70	8.39	8.19
TEMP	13.59	33.28	26.94	23.67
**Pond 2**	**Min**	**Max**	**Med**	**Mean**
CHL	0.22	12.67	3.90	4.26
FDOM	0.25	35.73	11.29	12.72
DO	6.01	19.39	11.11	11.58
SPC	1.10	282.70	216.45	215.71
Phyco	0.37	127.69	4.91	6.95
NTU	4.02	924.85	28.48	38.62
pH	4.02	10.40	9.13	8.96
TEMP	13.43	31.89	26.12	24.97
**Pond 3**	**Min**	**Max**	**Med**	**Mean**
CHL	0.48	20.81	2.43	3.78
FDOM	15.26	33.97	20.65	22.55
DO	5.15	20.47	9.17	9.67
SPC	175.00	254.50	199.00	203.56
Phyco	0.09	6.86	1.41	1.67
NTU	4.78	35.32	8.56	10.01
pH	7.07	10.01	8.17	8.33
TEMP	13.88	32.25	27.02	25.70
